# Supplementary material for: Limiting motorboat noise on coral reefs boosts fish reproductive success
Source: Nat Commun. 2022 May 20;13:2822. doi: 10.1038/s41467-022-30332-5 (PMC9123000; doi:10.1038/s41467-022-30332-5)
Supplement: Supplementary file 1 — Supplementary Information [file 41467_2022_30332_MOESM1_ESM.pdf]

Supplementary Information: Limiting motorboat noise on coral reefs boosts fish reproductive success

Field Study

Noise exposure regime

We experimentally elevated motorboat noise at three of the six sites (busy-boating treatment) to mimic typical traffic around a port, harbour or regularly visited reef. At these sites, we drove 5 m aluminium motorboats with 40 hp Suzuki four-stroke outboard engines repeatedly along the length of the site within 10–30 m of the edge of the reef. Busy-boating sites received an average of 180 motorboat passes each day during 3–6 'exposure periods' lasting 15–20 minutes each; this totalled 1.25–1.5 h per day of traffic noise at each busy-boating site. To minimise issues of pseudoreplication, eight different boats were driven by eight different drivers over the course of the experiment; drivers did not always drive the same boat. Boat speed varied from half to full throttle although was full throttle 90% of the time, representing passing traffic. Background traffic was present at a level of 0–10 passes per day from similar outboard motorboats with two- and four-stroke engines, and occasional passes by other larger (6 m hull, with single or twin 90 hp outboard motors; <1 per week) and smaller (3 m with one 5–20 hp outboard engine; ~1 per month) vessels. Busy-boating sites received motorboat activity on 85 days out of the 90-day season (except 25, 26, 27, 29 Dec, 1 Jan). Exposures occurred during daylight hours (06:30–18:00), the time when most motorboat traffic normally occurs. The total duration of boat driving in the experiment was 425 h. The other three sites were protected from motorboat traffic (limited-boating treatment). At limited-boating sites, motorboat drivers were recommended to avoid the sites by >100 m. When experimenters needed to access protected sites, speed was reduced to that where no wake was created (roughly ¼ throttle) within 100 m and boats were anchored 20 m from the reef. The primary source of motorboat traffic noise in the lagoon is research vessels; the island is otherwise uninhabited apart from a hotel on the other side of the island and non-research related traffic is present in the lagoon only rarely. We marked our limited-boating sites on a map in the research station and asked that they be kept quiet; this prevented other researchers using these sites. We monitored the lagoon daily to be confident that our limitation on boat traffic was effective.

Average background traffic for the whole lagoon area in the absence of any manipulations or recommendations was approximately 0–3 boats per day, dependent on site (see Chapter 4 and particularly Figure 4.6 in (*47*) for detailed descriptions of typical vessel traffic from the research station in the lagoon during the breeding season). Average background traffic at busy-boating sites was ~3 boats per day passing within 100 m and at limited-boating sites ~0.2 boats per day passing within 100 m.

Acoustic recordings and analysis

We made acoustic recordings in both pressure and particle-motion domains using an accelerometer with integrated hydrophone (M20-040 manufactured and calibrated by Geospectrum Techologies Inc. Dartmouth, Canada; sensitivity follows a curve from 0–5000 Hz) and a digital recorder (Zoom F4, Zoom Corporation, Tokyo, Japan; calibrated using pure sine waves measured with an oscilloscope). The accelerometer was attached by an elasticated line to a metal frame that sat on the bottom so that the instrument was 50 cm above the seabed. Sound recordings were analysed in *paPAM* (*40*). Recordings were low-pass filtered at 2 kHz before mean power spectral density levels (PSD) were calculated. Other damselfish are known to hear frequencies up to around 1200 Hz (*48*); we present acoustic data up to 2 kHz as the

hearing abilities of the focal species are currently unknown. Boat noise represented typical boat activity that the busy-boating sites experienced during the experiment. For PSDs, the window length was set equal to the sample rate (48 kHz) with a 50% overlap and a Hamming window, see Fig. 6.

Laboratory Study

Tank set up and conditions

We placed 25 female–male pairs of adult spiny chromis in 200-litre cylindrical tanks (one pair per tank; water depth 42 cm, diameter 60 cm). Pairs were fed twice daily (morning and afternoon) with approximately 0.45 g of O.range NRD G12 (1,200 µm) fish hatchery diet; a protein-based mix optimised for fish growth. Tanks contained half a terracotta pot for use as a shelter and an underwater loudspeaker mounted at the side, facing the shelter but not in contact with the side of the tank. Tanks were not aerated to minimise noise disturbance and were placed on rubber mats and coated in insulation foam to minimise the transmission of sound to neighbouring tanks.

All seawater was filtered at a flow rate of 10 L per minute with 100-micron bags, a UV steriliser, protein skimmer and biofilter. Experimental tanks were maintained at a mean±SD temperature of 27.7±0.6°C and placed under a 14:10-hour light:dark lighting regime.

Juveniles were fed twice daily (morning and afternoon) with approximately 0.35 g O.range WEAN-S (250 µm) fish hatchery diet.

We kept most of each brood with the parents to measure survival (cannibalism can rarely occur in this species under stress (*41*)) and isolated 50 individuals per brood as a single group in a separate tank (where parents could not compete with offspring for food) with the same playback treatment to measure growth. A further 50 individuals per brood were kept in similar conditions, but in the opposite sound treatment, for another study. Newly hatched broods were counted by taking the maximum count from three photos (mean±SE=203±56 hatchlings in busy-boating and 248±31 in no-boating). Offspring that were isolated from their parents were kept in one of four separate juvenile tanks in a 5-litre subdivision with other members of their own brood. The juvenile tanks were cylindrical (depth 40 cm, diameter 110 cm) and contained an underwater loudspeaker in the centre, facing upwards, with subdivisions arranged equidistant from the speaker around the edge of the tank.

Noise exposure regime

We played sound through loudspeakers (Lubell UW-30, University Sound, Whitehall, OH, USA; frequency range: 0.1–10 kHz) via MP3 players (SanDisk 8GB Clip Jam) and amplifiers (18 W, Kemo Langen Germany). Each MP3 player and amplifier powered two loudspeakers for the adult tanks, while the juvenile tanks had one mp3 player per loudspeaker. A battery pack (Cygnett Incharge 2500) was used to power each MP3 player, avoiding electrical interference from the main power supply.

We recorded ambient reef sounds during the day adjacent to healthy reefs at multiple inshore, shallow (~5 m), sandy-bottom locations around Lizard Island in 2013. We made motorboat-noise recordings at the same location, using one of five research station vessels (aluminium hull with 30 horsepower 4-stroke Suzuki outboard motors) driven at various speeds 10–200 m away, simulating boat traffic that may be experienced around coral reefs. Acoustic pressure was measured using a calibrated omnidirectional hydrophone (HiTech HTI-96-MIN with

inbuilt preamplifier; sensitivity flat across the frequency range 2 Hz – 30 kHz; -165 dB re 1 V/µPa; calibrated by manufacturers, High Tech Inc., Gulfport MS). Particle acceleration was measured using a triaxial accelerometer (Geospectrum M20-040; details above) and a digital recorder (Boss BR-800, 44.1 kHz sampling rate, Roland Corporation, Los Angeles, CA). Recordings were taken 1 m above the seabed.

We generated 'busy-boating' and 'no-boating' playbacks from field recordings. Pseudoreplication was minimised by randomly compiling ambient and boat sounds from different reefs and vessels, such that pairs of tanks were exposed to unique regimes that repeated once every nine days. The 'no-boating' playbacks were 12-hour sections of ambient reef sound (played during daylight hours: 06:00–18:00). The 'busy-boating' playbacks were identical with the addition of a pseudorandom number (minimum 3, maximum 6, 3-day average of 5) of 20-minute sections of boat noise spliced into each 12-hour section of ambient sound. The timing of boat-noise sections was random within daylight hours although overlapping more than one noise-exposure period within a playback was avoided. To avoid any potential impact of the soundtrack changing from one playback track type to another (such as any artefacts that could arise in the sounds as a result of combining files), the ambient playback had different ambient-sound sections with the same duration and timing spliced in to match the boat recordings in timing. All sound editing was completed using Audacity version 2.2.0 (http://www.audacityteam.org).

We measured acoustic pressure and particle acceleration of the playbacks in the experimental tanks (Fig. 6) using the same hydrophone and accelerometer as detailed above. The digital recorder used was different (Zoom F4, Zoom Corporation, Tokyo, Japan), but was calibrated in the same way (pure sine waves measured with an oscilloscope). The sound levels recorded in both the field and the experimental tanks were analysed using *paPAM* software (*46*). Power spectral density across the frequency range 0–2 kHz was calculated from 30-second clips for all treatments using Fast-Fourier Transformation. Boat-noise and ambient-sound levels from the field and tank are clearly separated in both acoustic pressure and particle acceleration plots (Fig. 6).

Table S1. Full model outputs for mixed effects models. Maximum likelihood ratio tests that employ chi-square statistics were used to compare models that contained or dropped each fixed effect.

**Model**	**Effect type**	**Effect name**	**Effect size ± SE**	***df***	***X^2***	***P***
**(A) Time in season clutches hatched (wild, 86 broods)**						
Linear mixed-effects model (LMM)	Intercept	NA				
	Fixed	Treatment	5.78 ± 5.34	1	1.23	0.268
	Random	Site	8.47 ± 2.91			
**(B) Number of predators of juveniles around nests over season (wild, 33 broods)**						
Negative binomial generalised linear	Intercept	NA				

mixed-effects model (GLMM)						
	Fixed	Treatment	0.68 ± 0.42	1	1.39	0.239
		Days	0.00 ± 0.02	1	1.49	0.223
	Random	Clutch	0.00 ± 0.00			
		Site	0.12 ± 0.34			
**(C) Juvenile survival (wild, 59 broods)**						
Cox survival model	Intercept	NA				
	Fixed	Treatment*Start count	0.004 ± 0.002	1	40.79	1.69×10^{-10}
	Fixed	Treatment	0.08 ± 0.65	1	34.87	3.52×10^{-9}
	Fixed	Start count	0.01 ± 0.001	1	260.72	$<2.2 \times 10^{-16}$
	Random	Clutch	0.28 ± 0.08			
		Site	0.75 ± 0.56			
**(D) Juvenile standard length (mm, day) (wild, 22 broods) (log transformed)**						
Linear mixed-effects model (LMM)	Intercept	NA				
	Fixed	Treatment*Age	$1.94 \times 10^{-3} \pm 8.99 \times 10^{-4}$	1	4.56	0.033
		Treatment	0.04 ± 0.03	1	1.05	0.305
		Age	$0.02 \pm 8.58 \times 10^{-4}$	1	543.54	$<2.2 \times 10^{-16}$
	Random	Clutch	$3.91 \times 10^{-3} \pm 0.07$			
		Site	$3.14 \times 10^{-4} \pm 0.18$			
**(E) Juvenile standard length (mm) day 21 and 42 post hatch (laboratory, 19 clutches)**						
Linear mixed-effects model (LMM)	Intercept	NA				
	Fixed	Treatment*Age	0.04 ± 0.01	1	7.95	0.005
		Treatment	0.40 ± 0.79	1	2.10	0.147

		Age	0.37 ± 0.01	1	714.12	<2.2 x 10^{-16}
	Random	Clutch	1.70 ± 1.30			
**(F) Juvenile dry weight (mg) day 21 and 42 post hatch (laboratory, 19 clutches)**						
Linear mixed-effects model (LMM)	Intercept	NA				
	Fixed	Treatment*Age	0.01 ± 0.01	1	1.58	0.209
		Treatment	0.09 ± 0.26	1	0.12	0.730
		Age	0.07 ± 0.00	1	340.55	<2.2 x 10^{-16}
	Random	Clutch	0.28 ± 0.53			
**(G) Egg area (mm^2) at laying (laboratory, 22 clutches)**						
Linear mixed-effects model (LMM)	Intercept	NA				
	Fixed	Treatment	0.01 ± 0.20	1	7 x 10^{-4}	0.978
	Random	Clutch	0.30 ± 0.55			
**(H) Yolk sac area (mm^2) at laying (laboratory, 22 clutches)**						
Linear mixed-effects model (LMM)	Intercept	NA				
	Fixed	Treatment	0.004 ± 0.20	1	6 x 10^{-4}	0.981
	Random	Clutch	0.21 ± 0.45			
**(I) Dry egg weight (mg) at laying (laboratory, 22 clutches)**						
Linear mixed-effects model (LMM)	Intercept	NA				
	Fixed	Treatment	1.46 ± 0.01	1	0.11	0.735
	Random	Clutch	0.01 ± 0.12			
**(J) Egg area (mm2) during embryonic development (laboratory, 22 clutches)**						
Linear mixed-effects model (LMM)	Intercept	NA				
	Fixed	Treatment*Age	0.05 ± 0.01	1	33.69	6.47 x 10^{-9}
		Treatment	0.02 ± 0.13	1	2.86	0.091

		Age	0.06 ± 0.01	1	337.54	<2.2 x 10^{-16}
	Random	Clutch	0.07 ± 0.27			
**(K) Embryo spine length (mm) day 10 development (laboratory, 22 clutches)**						
Linear mixed-effects model (LMM)	Intercept	NA				
	Fixed	Treatment	0.23 ± 0.08	1	8.10	0.004
	Random	Clutch	0.02 ± 0.15			
**(L) Yolk sac cross-sectional area (mm^2) day 10 development (laboratory, 22 clutches)**						
Linear mixed-effects model (LMM)	Intercept	NA				
	Fixed	Treatment	0.30 ± 0.08	1	11.19	8.24 x 10^{-4}
	Random	Clutch	0.03 ± 0.17			
