# Supplementary material for: Limiting motorboat noise on coral reefs boosts fish reproductive success
Source: Nat Commun. 2022 May 20;13:2822. doi: 10.1038/s41467-022-30332-5 (PMC9123000; doi:10.1038/s41467-022-30332-5)
Supplement: Supplementary file 3 — Reporting Summary [file 41467_2022_30332_MOESM3_ESM.pdf]

nature portfolio

Corresponding author(s): Sophie Nedelec

Last updated by author(s): Apr 11, 2022

Reporting Summary

Nature Portfolio wishes to improve the reproducibility of the work that we publish. This form provides structure for consistency and transparency in reporting. For further information on Nature Portfolio policies, see our Editorial Policies and the Editorial Policy Checklist.

Statistics

For all statistical analyses, confirm that the following items are present in the figure legend, table legend, main text, or Methods section.

n/a	Confirmed	
☐	☒	The exact sample size (*n*) for each experimental group/condition, given as a discrete number and unit of measurement
☐	☒	A statement on whether measurements were taken from distinct samples or whether the same sample was measured repeatedly
☐	☒	The statistical test(s) used AND whether they are one- or two-sided<br>*Only common tests should be described solely by name; describe more complex techniques in the Methods section.*
☐	☒	A description of all covariates tested
☐	☒	A description of any assumptions or corrections, such as tests of normality and adjustment for multiple comparisons
☐	☒	A full description of the statistical parameters including central tendency (e.g. means) or other basic estimates (e.g. regression coefficient) AND variation (e.g. standard deviation) or associated estimates of uncertainty (e.g. confidence intervals)
☐	☒	For null hypothesis testing, the test statistic (e.g. *F*, *t*, *r*) with confidence intervals, effect sizes, degrees of freedom and *P* value noted<br>*Give P values as exact values whenever suitable.*
☒	☐	For Bayesian analysis, information on the choice of priors and Markov chain Monte Carlo settings
☒	☐	For hierarchical and complex designs, identification of the appropriate level for tests and full reporting of outcomes
☐	☒	Estimates of effect sizes (e.g. Cohen's *d*, Pearson's *r*), indicating how they were calculated

Our web collection on statistics for biologists contains articles on many of the points above.

Software and code

Policy information about availability of computer code

Data collection	BORIS 7.6.1 was used to identify predators around nests from videos. ImageJ 1.5.2d was used to generate manual tracking data from videos of fish activity. The distance traveled was calculated by marking the position of the fish every second using the manual tracking feature of ImageJ version 1.52d (https://imagej.nih.gov/ij/download.html). Fanning was observed from the side camera and analysed using Solomon Coder software (https://solomoncoder.com/download.php).
Data analysis	RStudio 1.4.1717 was used with standard statistical packages as described in the paper. paPAM 0.902 using MATLAB compiler runtime R2017bx64 https://gitlab.com/RTbecard/paPAM was used for sound anaalysis.

For manuscripts utilizing custom algorithms or software that are central to the research but not yet described in published literature, software must be made available to editors and reviewers. We strongly encourage code deposition in a community repository (e.g. GitHub). See the Nature Portfolio guidelines for submitting code & software for further information.

Data

Policy information about availability of data

All manuscripts must include a data availability statement. This statement should provide the following information, where applicable:

- Accession codes, unique identifiers, or web links for publicly available datasets
- A description of any restrictions on data availability
- For clinical datasets or third party data, please ensure that the statement adheres to our policy

All data are provided in the Source Data file provided with the paper. A section describing availability of data is in the methods: Data availability
All data are provided with this paper as a Source Data file named 'Nedelec et al 2022 Limited motorboat noise on coral reefs boost fish reproductive success Source Data.xlsx'.

Field-specific reporting

Please select the one below that is the best fit for your research. If you are not sure, read the appropriate sections before making your selection.

☒ Life sciences ☐ Behavioural & social sciences ☐ Ecological, evolutionary & environmental sciences

For a reference copy of the document with all sections, see nature.com/documents/nr-reporting-summary-flat.pdf

Life sciences study design

All studies must disclose on these points even when the disclosure is negative.

Sample size	Across the study, there were several layers of sample size. At the largest scale (coral reefs), we were restricted to what was feasible (3 reefs per treatment), but we made sure that reefs were long enough to include breeding adults for a sufficient sample size of broods (40/46 per treatment). For laboratory experiments, sample size of was determined by balancing the number of animals to be taken from the wild with a large enough sample size to indicate a difference if one was present. Sampling from clutches in the laboratory was decided upon to balance the largest sample size possible while ensuring enough offspring for sampling at later stages. We could not perform a power analysis because we did not have prior knowledge of likely effect sizes. Other experiments on our study species and other related species at MARFU informed our choice of sample size that would likely identify biologically meaningful differences.
Data exclusions	Three nests were excluded from counts of survival of offspring where counts increased due to assumed experimenter error or immigration (immigration is unlikely due to the territorial nature of parents defending offspring). This is stated in the methods of the paper.
Replication	We conducted one field study and one laboratory study. The laboratory study was performed to replicate effects seen in the wild, to ensure that the effects observed were due to noise and no other factor.
Randomization	Treatments were allocated to field sites partly randomly and partly allowing for ease and safety of motorboat access. Allocation of treatments to tanks in the laboratory was random.
Blinding	Lead investigator SLN allocated laboratory tanks to treatments using a random sequence generator before seeing the tanks or the fish involved in the study.<br><br>Field sites were initially randomly allocated to treatments, after which, two sites were switched in treatment to allow safety of motorboat access by multiple drivers.<br><br>During video and photo analysis, observers were made blind to the treatment by cryptic labeling.

Reporting for specific materials, systems and methods

We require information from authors about some types of materials, experimental systems and methods used in many studies. Here, indicate whether each material, system or method listed is relevant to your study. If you are not sure if a list item applies to your research, read the appropriate section before selecting a response.

Materials & experimental systems

n/a	Involved in the study	
☒	☐	Antibodies
☒	☐	Eukaryotic cell lines
☒	☐	Palaeontology and archaeology
☐	☒	Animals and other organisms
☒	☐	Human research participants
☒	☐	Clinical data
☒	☐	Dual use research of concern

Methods

n/a	Involved in the study	
☒	☐	ChIP-seq
☒	☐	Flow cytometry
☒	☐	MRI-based neuroimaging

Animals and other organisms

Policy information about studies involving animals; ARRIVE guidelines recommended for reporting animal research

Laboratory animals	Study did not use laboratory animals.
Wild animals	Animals observed in the field: adult and juvenile spiny chromis (Acanthochromis polyacanthus). For the laboratory study, spiny chromis adults were caught with barrier nets and hand nets from shallow reefs around Lizard Island in the northern Great Barrier Reef during November 2016. Spiny chromis were transported in seawater in polystyrene containers and housed in 25 male-female pairs at MARFU. The age of adults used for laboratory study taken from the wild was unknown but all were adults (>1yr) and similar in size (The mean and SE standard length of adults was 10.7 +/-.1cm). To measure the growth of offspring we removed a subset of individuals from some broods. Offspring were transported from the field site to the field station in bags of fresh seawater and

following standard established practice were euthanised using an ice slurry for immediate full colour photography. Offspring were euthanised to avoid the extreme stress and inevitable mortality of removal from their brood and parental care, measurement procedures and re-release. Adults were kept at MARFU after the end of the experiment for further experiments.

Field-collected samples

We conducted the laboratory study in the Marine and Aquaculture Research Facilities Unit (MARFU) at James Cook University, Townsville, Australia from March to July 2018. Spiny chromis adults were caught with barrier nets and hand nets from shallow reefs around Lizard Island in the northern Great Barrier Reef (14° 41′ S, 145° 27′ E) during November 2016. The mean±SE standard length of adults was 10.7±0.1 cm. Spiny chromis were housed in 25 male–female pairs, and maintained at a mean±SE temperature of 27.7 ±0.1°C in the presence of either a busy-boating treatment (playback of motorboats in a pattern matching exposures in the field) or a no-boating treatment (playback of ambient reef sound). We kept most of each brood with the parents to measure survival (cannibalism can rarely occur in this species under stress) and isolated 50 individuals per brood as a single group in a separate tank (where parents could not compete with offspring for food) with the same playback treatment to measure growth.
Tank set up and conditions
We placed 25 female–male pairs of adult spiny chromis in 200-litre cylindrical tanks (one pair per tank; water depth 42 cm, diameter 60 cm). Pairs were fed twice daily (morning and afternoon) with approximately 0.45 g of O.range NRD G12 (1,200 μm) fish hatchery3
nature research | reporting summary April 2020
diet; a protein-based mix optimised for fish growth. Tanks contained half a terracotta pot for use as a shelter and an underwater loudspeaker mounted at the side, facing the shelter but not in contact with the side of the tank. Tanks were not aerated to minimise noise disturbance and were placed on rubber mats and coated in insulation foam to minimise the transmission of sound to neighbouring tanks.
All seawater was filtered at a flow rate of 10 L per minute with 100-micron bags, a UV steriliser, protein skimmer and biofilter. Experimental tanks were maintained at a mean±SD temperature of 27.7±0.6°C and placed under a 14:10-hour light:dark lighting regime.
Juveniles were fed twice daily (morning and afternoon) with approximately 0.35 g O.range WEAN-S (250 μm) fish hatchery diet.
We kept most of each brood with the parents to measure survival (cannibalism can rarely occur in this species under stress (32)) and isolated 50 individuals per brood as a single group in a separate tank (where parents could not compete with offspring for food) with the same playback treatment to measure growth. A further 50 individuals per brood were kept in similar conditions, but in the opposite sound treatment, for another study. Newly hatched broods were counted by taking the maximum count from three photos (mean±SE=203±56 hatchlings in busy-boating and 248±31 in no-boating). Offspring that were isolated from their parents were kept in one of four separate juvenile tanks in a 5-litre subdivision with other members of their own brood. The juvenile tanks were cylindrical (depth 40 cm, diameter 110 cm) and contained an underwater loudspeaker in the centre, facing upwards, with subdivisions arranged equidistant from the speaker around the edge of the tank.

Ethics oversight

University of Exeter Biociences Streatham Ethics Committee. James Cook University Animal Ethics Committee.

Note that full information on the approval of the study protocol must also be provided in the manuscript.
